# Publisher Correction: Joint encryption and error correction for secure quantum communication

**DOI:** 10.1038/s41598-024-77826-4

**Published:** 2024-10-29

**Authors:** Nitin Jha, Abhishek Parakh, Mahadevan Subramaniam

**Affiliations:** 1 Kennesaw State University, Kennesaw, GA USA; 2 University of Nebraska Omaha, Omaha, NE USA

Correction to: *Scientific Reports* 10.1038/s41598-024-75212-8, published online 19 October 2024

The Funding section in the original version of this Article was omitted.

The Funding section now reads: "This work is sponsored by NSF award numbers 2324924 and 2324925."

The original Article has been corrected.

